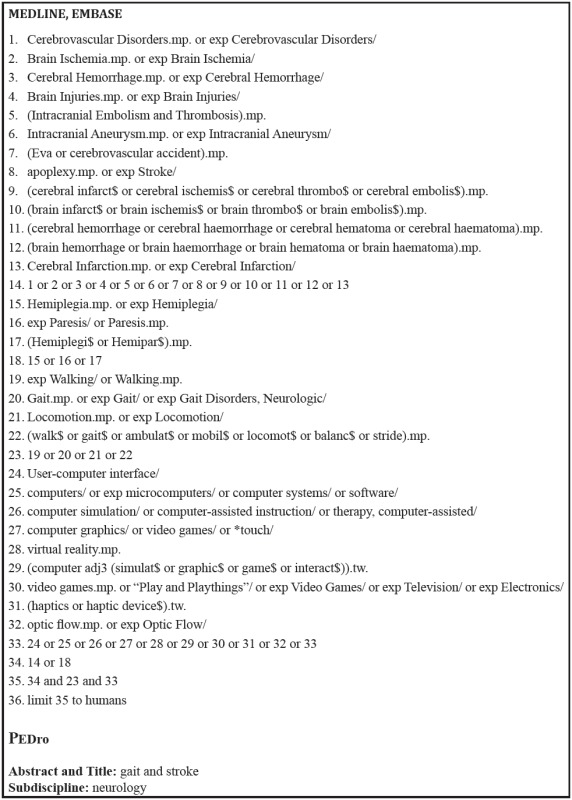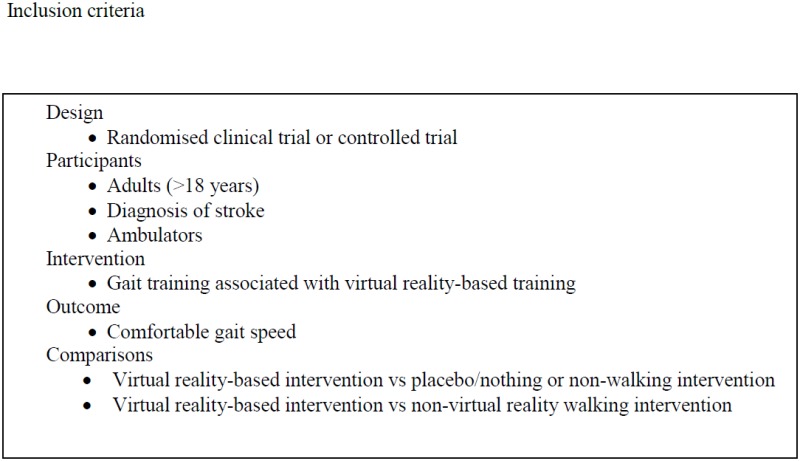# Walking training associated with virtual reality-based training increases walking speed of individuals with chronic stroke: systematic review with meta-analysis

**DOI:** 10.1590/bjpt-rbf.2014.0062

**Published:** 2014

**Authors:** Juliana M. Rodrigues-Baroni, Lucas R. Nascimento, Louise Ada, Luci F. Teixeira-Salmela

**Affiliations:** 1 Rede Sarah de Hospitais de Reabilitação, Belo Horizonte, MG, Brazil; 2 Discipline of Physiotherapy, Faculty of Health Sciences, The University of Sydney, Sydney (NSW), Australia; 3 Departamento de Fisioterapia, Universidade Federal de Minas Gerais (UFMG), Belo Horizonte, MG, Brazil

## Abstract

**OBJECTIVE:**

To systematically review the available evidence on the efficacy of walking training associated with virtual reality-based training in patients with stroke. The specific questions were: Is walking training associated with virtual reality-based training effective in increasing walking speed after stroke? Is this type of intervention more effective in increasing walking speed, than non-virtual reality-based walking interventions?

**METHOD:**

A systematic review with meta-analysis of randomized clinical trials was conducted. Participants were adults with chronic stroke and the experimental intervention was walking training associated with virtual reality-based training to increase walking speed. The outcome data regarding walking speed were extracted from the eligible trials and were combined using a meta-analysis approach.

**RESULTS:**

Seven trials representing eight comparisons were included in this systematic review. Overall, the virtual reality-based training increased walking speed by 0.17 m/s (IC 95% 0.08 to 0.26), compared with placebo/nothing or non-walking interventions. In addition, the virtual reality-based training increased walking speed by 0.15 m/s (IC 95% 0.05 to 0.24), compared with non-virtual reality walking interventions.

**CONCLUSIONS:**

This review provided evidence that walking training associated with virtual reality-based training was effective in increasing walking speed after stroke, and resulted in better results than non-virtual reality interventions.

## Introduction

After stroke, individuals often exhibit motor impairments, which are associated with activity limitations and social participation restrictions. Walking limitations is one of the main causes of disabilities after stroke, as the ability to walk is directly related to functional independence^1^^,^ 2. According to Alzahrani et al.^3^, if walking performance is poor after stroke, activities at home and in the community will be limited, so that people may become housebound and isolated from society.

The mean walking speed after stroke varies from 0.4 to 0.8 m/s^4^^-^ 6. Walking speeds of less than 0.4 m/s define household ambulation; speeds between 0.4 and 0.8 m/s define limited community ambulation; and speeds greater than 0.8 m/s define full community ambulation. Consequently, a significant focus of interest in rehabilitation trials is to identify the effectiveness of interventions, which are able to increase walking speed after stroke, as greater speed is related to improved social participation and quality of life^3^^,^ 4. Although previous systematic reviews have evidenced the efficacy of both overground and treadmill training in improving walking speed^5^^-^ 7, new techniques and instruments can be added to usual walking training, to optimize the effect of interventions aimed at improving walking ability after stroke.

Some studies have suggested that virtual reality might be a useful tool in the rehabilitation of individuals after stroke, and its effect on walking speed have started being investigated^8^^-^ 11. By definition, virtual reality is the use of interactive simulations created with computer hardware and software to provide users with opportunities to engage in environments that appear and feel similar to real-world objects and events^5^. A wide variety of interfaces that allow the interactions with virtual environments is currently available. Components may be common devices, such as mouse, keyboards or joysticks, or more complex systems with cameras, sensors, and feedback devices, providing the users with the sensation of touching targets or deviating from objects, which are similar to obstacles present in the real world^11^^,^ 12.

According to Dobkin^13^, the addition of virtual reality elements to walking interventions is advantageous, as it provides training in an enriched environment similar to the real environment patients experience in daily life. In addition, virtual tasks have been described as more interesting and enjoyable by both children and adults, thereby, encouraging more time of practice and higher number of repetitions, which are considered to be important factors in the rehabilitation of individuals with neurologic disorders^8^^,^ 14. Concerning walking rehabilitation, the use of virtual environments enables therapists to progressively modulate the levels of difficulty of the tasks to challenge patients and to provide them with immediate feedback regarding their performance. Furthermore, clinicians are able to train tasks that are unsafe to practice in the real world, such as overcoming obstacles or crossing streets^8^^,^ 13.

Two previous systematic reviews have examined the effect of walking training associated with virtual reality-based training in improving walking ability after stroke. A *Cochrane* 8 review reported a non-significant increase in walking speed of 0.07 m/s (95% CI:-0.09 to 0.23), based upon three randomised clinical trials. A more recent review^9^ included four randomised clinical trials and indicated that the addition of virtual reality-based training was beneficial to walking ability after stroke. However, the authors reported clinical heterogeneity between the trials, and a meta-analysis was not performed. Therefore, the results regarding the addition of virtual reality-based training to walking interventions aimed at improving walking ability after stroke remain inconclusive. In addition, there were not found any reviews that separately examined the efficacy of walking training associated with virtual reality-based training and the superiority of this association, compared with other walking interventions.

Therefore, the aim of this systematic review was to examine the effect of the addition of virtual reality-based training to walking training for improving walking speed after stroke. The specific research questions were:

1. Is walking training associated with virtual reality-based training effective in increasing walking speed after stroke?
2. Is walking training associated with virtual reality-based training more effective than non-virtual reality-based interventions?

In order to make recommendations based upon a high level of evidence, this review planned to include only randomised or controlled trials.

## Method

### Identification and selection of trials

Searches were conducted at the MEDLINE (1946 to July 2013), PEDro (to July 2013), and EMBASE (1980 to July 2013) databases for relevant studies without language restrictions. Search terms included words related to *stroke*, *virtual reality training* (such as virtual reality, video-games, flow optic) and *gait* (Appendix 1). Titles and abstracts were displayed and screened by one reviewer to identify relevant studies. Full paper copies of relevant peer-reviewed papers were retrieved and their reference lists were also screened to identify further relevant studies. The selection of the retrieved papers was conducted by two reviewers, using predetermined criteria, which are summarized in the supplementary materials related to this paper (Appendix 1S^[1])^.

### Assessment of characteristics of the trials

**Quality** **:** The quality of the included trials was assessed by extracting PEDro scores from the Physiotherapy Evidence Database^15^. PEDro is an 11-item scale designed for rating the methodological quality (internal validity and statistical information) of randomised trials. Each item, except for Item 1, contributes one point to the total score (range: 0 to 10 points). Where a trial was not included on the database, it was scored by a reviewer, who had completed the PEDro scale training tutorial.

**Participants** **:** Trials involving ambulatory adults after stroke were included. The number of participants, age, time since stroke, and baseline walking speed were recorded to assess the similarity of the studies.

**Intervention** **:** The experimental intervention was walking training associated with virtual reality-based training aimed at improving walking speed after stroke. Virtual reality was defined as a simulation of a real environment created by a computer software which allowed users to interact with elements within a simulated scenario by using different interfaces, such as mouse, keyboards, joysticks, gloves, and/or motion capture systems^11^^,^ 12. We included trials using any form of non-immersive or immersive virtual reality, and those that used commercially available gaming consoles^8^.

The control intervention was defined according to the research questions: (i) to examine the efficacy of walking training associated with virtual reality-based training, the control intervention could be nothing/placebo or any other non-walking intervention; (ii) to examine the superiority of walking training associated with virtual reality-based training, the control intervention could be any other non-virtual reality walking intervention.

**Outcome measure** **:** The outcome measure of interest was comfortable walking speed, provided, in this review, in meters per second (m/s). The timing of the measurements and the procedure used to measure walking speed were recorded to assess the appropriateness of combining the studies in a meta-analysis.

### Data analysis

Information about the method (i.e., design, participants, interventions, and outcome measures) and results (i.e., number of participants, and means (SD) of walking speed) were extracted by one reviewer and checked by a second one. Where information was not available in the published trials, details were requested from the corresponding author.

The post-intervention scores were used to obtain the pooled estimate of the effect of intervention. The effect size was obtained using the fixed effects model and reported as weighted mean differences (MD) with 95% confidence intervals (95% CI)**.** In the case of significant statistical heterogeneity (I^2^>50%), a random effects model was applied to check the robustness of the results. The analyses were performed using the MIX-Meta-Analysis Made Easy program Version 1.7^16^^,^ 17; the significance level for statistical heterogeneity was set at 5% (two-tailed). Where data were not available to be included in the pooled analysis, the between-group results were reported.

## Results

### Flow of trials through the review

**Figure 1:**
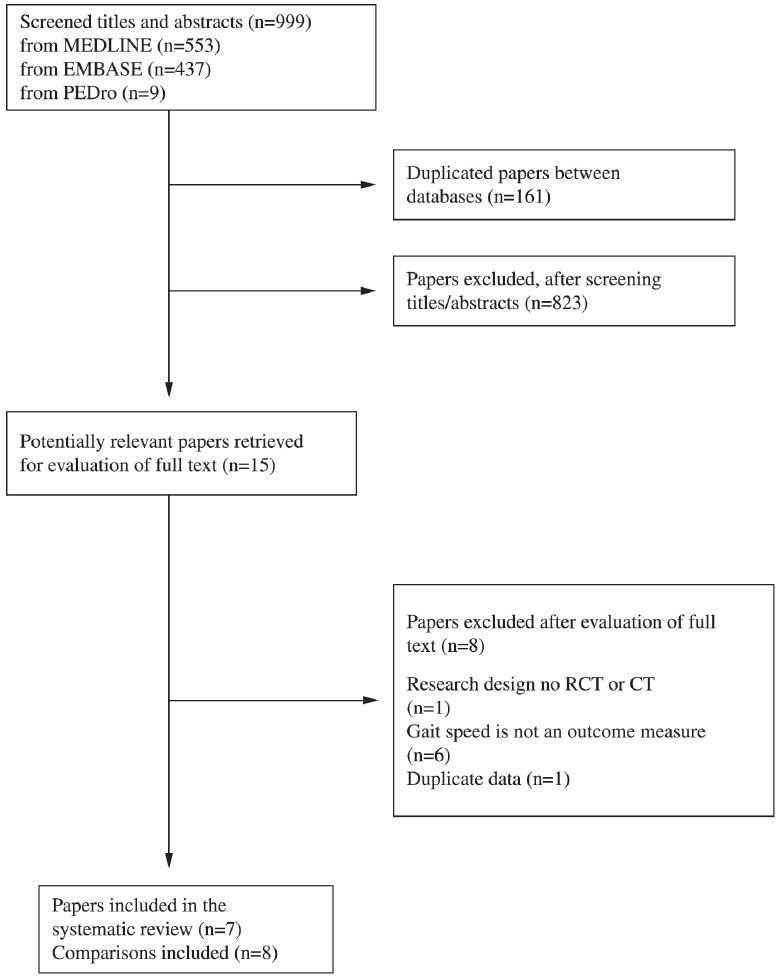
Flow of studies through the review. RCT = randomised clinical trial; CT = controlled trial.

The electronic search strategy identified 999 relevant papers for the analysis of titles and abstracts. After screening titles and abstracts, 15 potentially relevant full papers to answer the research questions were retrieved. Following the analysis, according to the predetermined inclusion criteria, eight papers were retrieved. After data extraction, one paper^18^ was removed from the review, because its results included duplicate data of a second paper^19^. Therefore, seven papers were included in this review (Figure 1).

### Characteristics of the included trials

Seven randomized clinical trials involving 154 participants examined the efficacy of walking training associated with virtual reality-based training for increasing walking speed after stroke, and therefore were included in this review (Table 1). Since one of the trials^20^ included two control groups, a total of eight comparisons were performed. Three trials^20^^-^ 22 compared walking training associated with virtual reality-based training with nothing/placebo or non-walking intervention (Question 1). Five trials^19^^,^ 20 ^,^ 23 ^-^ 25 compared walking training associated with virtual reality-based training with a non-virtual reality walking intervention (Question 2).

**Quality** **:** The mean PEDro score of the included trials was 6.1, ranging from 4 to 8 points (Table 2). All the trials randomly allocated participants, had similar groups at baseline, and reported point estimate and variability. The majority of trials reported concealed allocation (57%), had less than 15% drop-outs (57%), reported between-group differences (86%), and had blinded assessors (86%). However, the majority of trials did not report whether and intention-to-treat analysis was undertaken (86%). Only one trial^22^ blinded participants, and no trials blinded therapists, which is considered difficult or impossible during complex interventions.

**Participants :** The mean age of participants ranged from 52 to 66 years across trials. All trials included participants with time after stroke greater than six months (ranging from 10 to 72 months across trials), which defines chronic hemiparesis. The sample size of the included trials ranged between 14 and 30 participants, who were allocated to the experimental or control groups. All of the participants were ambulatory adults at the time of entry into the trial, with mean baseline walking speed ranging from 0.46 to 0.70 m/s across trials.

**Intervention :** In all trials, the experimental intervention was walking training associated with virtual reality-based training. Virtual reality-based training was associated with treadmill training in four trials^20^^,^ 23 ^-^ 25, with video-games exercises in two trials^21^^,^ 22, and with kinesiotherapy involving specific ankle movements in one trial^19^. Three trials^20^^,^ 22 ^,^ 23 delivered usual therapy to both experimental and control groups.

**Table 1:** Characteristics of papers (n=7) included in the systematic review on the addition of virtual reality-based training after stroke.

**Study**	**Design**	**Participants**	**Intervention**	**Walking speed measurements (week)**
Cho and Lee^23^	RCT	n=14Age (yr): 65 (4)Time since stroke (mth): 10 (2)WS: 0.53 (0.17)	Exp = Virtual reality-based treadmill training30min x 3/wk x 6wkCon = Treadmill training30min x 3/wk x 6wkBoth = Usual therapy	0 and 6
Fritz et al.^21^	RCT	n=28Age (yr): 66 (10)Time since stroke (mth): 36 (35)WS: 0.57 (0.30)	Exp = Video-game exercises60min x 4/wk x 5wkCon = no intervention	0, 5 and 12
Jaffe et al.^24^	RCT	n=20Age (yr): 62 (10)Time since stroke (mth): 45 (29)WS: not reported	Exp = Stepping over virtual obstacles in a treadmill60min x 3/wk x 2wkCon = Stepping over foam obstacles in a hallway30min x 3/wk x 2wk	0, 2 and 4
Kang et al.^20^	RCT	n=30Age (yr): 56 (7)Time since stroke (mth): 14 (5)WS: 0.5 (0.2)	Exp = Virtual reality-based treadmill training30min x 3/ wk x 4 wkCon1 = Treadmill training30min x 3/ wk x 4 wkCon2 = stretching exercises30min x 3/ wk x 4 wkAll groups = Usual therapy	0 and 4
Kim et al.^22^	RCT	n=24Age (yr): 52 (8)Time since stroke (mth): 24 (9)WS: 0.46 (0.15)	Exp = Video-game exercises30min x 4/wk x 4wkCon = no interventionBoth = Usual therapy	0 and 4
Mirelman et al.^19^	RCT	n=18Age (yr): 62 (9)Time since stroke (mth): 48 (26)WS: 0.66 (0.27)	Exp = Ankle movements with targets and feedback provided by virtual reality60min x 3/wk x 4wkCon = Ankle movements without feedback provided by virtual reality60min x 3/wk x 4wk	0, 4 and 7
Yang et al.^25^	RCT	n=20Age (yr): 61 (11)Time since stroke (mth): 72 (87)WS: 0.70 (0.44)	Exp = Virtual reality-based treadmill training20min x 3/wk x 3wkCon = Treadmill training20min x 3/wk x 3wk	0, 3 and 7

# groups and outcome measures listed are those which were analysed in this systematic review, there may have been other groups or measures in the paper. RCT = randomised clinical trial, WS = walking speed at baseline (m/s), Exp = experimental group, Con = control group.

The majority of trials delivered immersive virtual reality training to the experimental group. In these trials^20^^,^ 23 ^-^ 25, virtual images were coupled to the treadmill, and treadmill speed was changed as a function of the generated visual images. Non-immersive virtual reality was used in three trials^19^^,^ 21 ^,^ 22: two^21^^,^ 22 employed video cameras to capture the patient's body image and to enable interactions with virtual objects; one trial^19^ employed visual feedback on the computer screen and tactile feedback related to the patient's movements. Only one trial^21^ used a commercially available virtual reality device (*Nintendo Wii*) during the delivery of the experimental intervention.

**Table 2:** *PEDro *criteria and scores for the papers (n=7) included in the systematic review on the addition of virtual reality-based training after stroke.

**Study**	**Random allocation**	**Concealed allocation**	**Groups similar at baseline**	**Participant blinding**	**Therapist blinding**	**Assessor blinding**	**<15% dropouts**	**Intention-to-treat analysis**	**Between-group difference reported**	**Point estimate and variability reported**	**Total (0 to 10)**
Cho and Lee^23^	Y	Y	Y	N	N	Y	Y	N	Y	Y	7
Fritz et al.^21^	Y	Y	Y	N	N	Y	Y	Y	Y	Y	8
Jaffe et al.^24^	Y	N	Y	N	N	N	Y	N	N	Y	4
Kang et al.^20^	Y	Y	Y	N	N	Y	Y	N	Y	Y	7
Kim et al.^22^	Y	N	Y	Y	N	Y	N	N	Y	Y	6
Mirelman et al.^19^	Y	N	Y	N	N	Y	N	N	Y	Y	5
Yang et al.^25^	Y	Y	Y	N	N	Y	N	N	Y	Y	6

Y= yes; N=no.

**Outcome measures:** The majority of trials used a timed walk measure based upon the 10-Meter Walk Test^26^ to measure walking speed, with variations on the length of the corridor: 12^25^, 10^20^^,^ 22, seven^19^, six^24^, and three meters^21^. One trial^23^ used foot sensors during a timed walk test from a specific device (GAITRite; CIR System Inc, New Jersey) to measure walking speed. All the data in this review reflects comfortable gait speed and were converted to m/s.

### Effect of walking training associated with virtual reality-based training on walking speed

**Figure 2:**
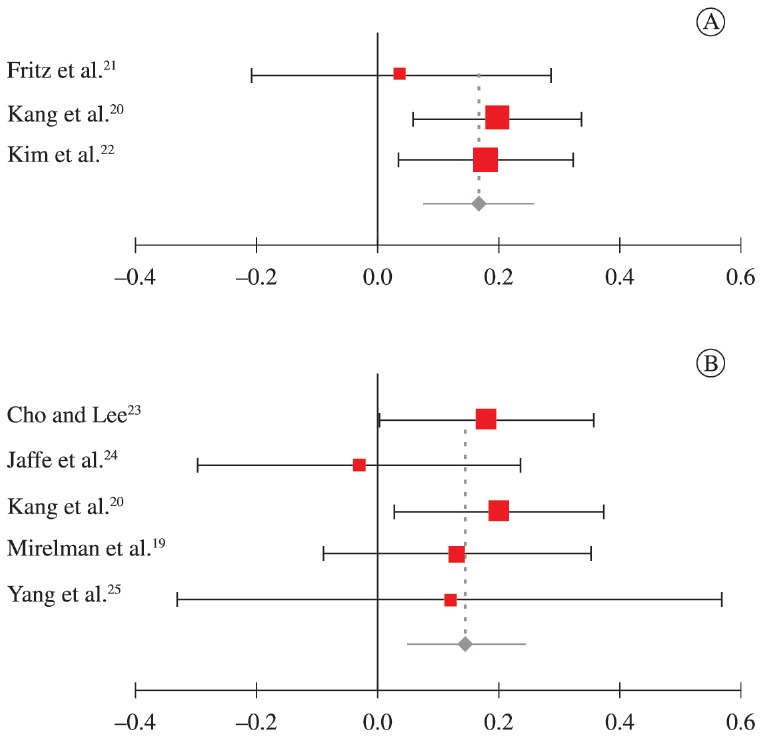
A. Mean difference (95% CI) of the effect of virtual reality-based intervention versus nothing/placebo or non-walking intervention on walking speed immediately after intervention (n=72). **B**. Mean difference (95% CI) of the effect of virtual realitybased intervention versus non-virtual reality walking intervention on walking speed immediately after intervention (n=92).

The overall effect of walking training associated with virtual reality-based training on walking speed immediately after intervention was examined by pooling post-intervention data from three trials^20^^-^ 22 with a mean PEDro score of 7.0, indicating good quality^27^. Virtual reality-based training increased walking speed by 0.17 m/s (95% CI 0.08 to 0.26; *fixed effects model* I^2^=0%), compared with placebo/nothing or non-walking interventions (Figure 2A).

### Effect of walking training associated with virtual reality-based training, compared with non-virtual reality walking interventions on walking speed

The superiority of walking training associated with virtual reality-based training on walking speed immediately after intervention was examined by pooling post-intervention data from five trials^19^^,^ 20 ^,^ 23 ^-^ 25 with a mean PEDro score of 5.8, indicating moderate quality^27^. Virtual reality-based training increased walking speed by 0.15 m/s (95% CI 0.05 to 0.24; *fixed effects model* I^2^=0%), compared with non-virtual reality walking interventions (Figure 2B).

## Discussion

This systematic review provided clinical evidence that walking training associated with virtual reality-based training was effective in increasing walking speed after stroke. Clinically, the results indicated that the addition of virtual reality-based training is more effective than no intervention, placebo, or non-walking interventions. The results also indicated that walking training associated with virtual reality-based training produced faster walking speed, compared with non-virtual reality walking interventions.

The meta-analysis demonstrated that the addition of virtual reality-based training increased walking speed by 0.17 m/s. This meta-analysis was the first to examine the efficacy of this type of intervention to improve walking speed with individuals after stroke. Importantly, these benefits appear to be clinically meaningful. For example, Tilson et al.^28^ demonstrated that a between-group difference in walking speed after stroke greater than 0.16 m/s resulted in improvement in the patients' levels of disability, and suggested this value as a rehabilitation goal. The meta-analysis also demonstrated that walking training associated with virtual reality-based training produced 0.15 m/s faster walking, than other non-virtual walking interventions. A previous systematic review^8^ have reported a non-significant between-group difference after the addition of virtual reality-based training. The inclusion of two extra clinical trials in the present meta-analysis increased its statistical power and strengthens the evidence the efficacy of the addition of virtual reality-based training for increasing walking speed after stroke.

This review examined the effect of the addition of virtual reality-based training to various types of walking intervention after stroke. Although various types of walking training have been employed across trials (i.e., treadmill training^20^^,^ 23 ^-^ 25, exercises using videogames^21^^,^ 22, or ankle exercises^19^), overall, the included trials were similar in terms of session duration (mean 41 min, SD 18), session frequency (mean 3.3/wk, SD 0.5), program duration (4 weeks, SD 1), participants' characteristics, and aim of intervention. In addition, statistical analysis (I^2^=0%) indicated that the trials were clinical and statistically similar, which allowed for the data to be combined in meta-analyses. The data suggested similarity across trials and indicated lack of clinical or statistical heterogeneity, supporting the clinical evidence that the addition of virtual reality-based training is effective in improving walking speed after stroke.

Although the improvement in walking speed was superior with the addition of virtual reality-based training, other factors not examined in this review, such as clients' values and expectations, clinical expertise, and costs of implementation should be taken into consideration before deciding the most appropriate type of intervention for each client. The gaming industry has recently released low-cost virtual reality systems, such as *Nintendo Wii*, *Kinect,* and *Playstation*, thus facilitating the access of rehabilitation centers and home users to this technology^29^^,^ 30. However, only one trial^21^ included in this review used commercially available devices, and the between-group difference was not clinically significant for walking speed (mean difference: 0.04 m/s, 95% CI:-0.22 to 0.30). Subgroup analysis based upon the type of virtual reality delivered could not be performed, because there were not enough trials. Thus, new clinical trials examining the efficacy of the addition of virtual reality-based training using commercially available devices are encouraged.

This review has both strengthens and limitations. A source of bias in the included trials was lack of blinding of therapists and participants, since it is very difficult or unpractical to blind either during the delivery of complex interventions, such as walking training. In addition, the majority of the included trials did not report whether an intention-to-treat analysis was carried-out. On the other hand, the mean PEDro score of 6.1 for the included trials indicated good methodological quality^27^. One second positive aspect was the inclusion of trials that examined the same outcome measure - walking speed; this allowed the exhibition of results in weighted mean difference, which is clinically intelligible. Furthermore, the inclusion of only trials whose intervention was walking training associated with virtual reality-based training constraints the results to a specific intervention.

## Conclusions

This systematic review provided clinical evidence for the efficacy of the addition of virtual reality-based training to walking training in improving walking speed after stroke, compared with placebo or no intervention. In addition, this review demonstrated that walking training associated with virtual reality-based training was more effective in improving walking speed, compared with non-virtual reality walking interventions. The results are based on a meta-analysis of seven randomized clinical trials with good methodological quality. Clinicians should, therefore, be confident in prescribing walking training associated with virtual reality-based training to improve walking speed after stroke. Other factors, such as clients' values and expectations, clinical expertise, and costs of implementation should also be considered, when deciding on the most appropriate type of intervention for each client.

## Appendix 1 Search strategy for the systematic review on the addition of virtual reality-based training after stroke.